# Impact of past and on-going changes on climate and weather on vector-borne diseases transmission: a look at the evidence

**DOI:** 10.1186/s40249-019-0565-1

**Published:** 2019-06-13

**Authors:** Florence Fouque, John C. Reeder

**Affiliations:** UNICEF/UNDP/ World Bank/WHO Special Programme for Research and Training in Tropical Diseases (TDR), 20 Avenue Appia, 1211 Geneva 27, Switzerland

**Keywords:** Climate change, Vector-borne diseases, Mosquitoes vectors, malaria, arboviruses

## Abstract

**Background:**

The climate variables that directly influence vector-borne diseases’ ecosystems are mainly temperature and rainfall. This is not only because the vectors bionomics are strongly dependent upon these variables, but also because most of the elements of the systems are impacted, such as the host behavior and development and the pathogen amplification. The impact of the climate changes on the transmission patterns of these diseases is not easily understood, since many confounding factors are acting together. Consequently, knowledge of these impacts is often based on hypothesis derived from mathematical models. Nevertheless, some direct evidences can be found for several vector-borne diseases.

**Main body:**

Evidences of the impact of climate change are available for malaria, arbovirus diseases such as dengue, and many other parasitic and viral diseases such as Rift Valley Fever, Japanese encephalitis, human African trypanosomiasis and leishmaniasis. The effect of temperature and rainfall change as well as extreme events, were found to be the main cause for outbreaks and are alarming the global community. Among the main driving factors, climate strongly influences the geographical distribution of insect vectors, which is rapidly changing due to climate change. Further, in both models and direct evidences, climate change is seen to be affecting vector-borne diseases more strikingly in fringe of different climatic areas often in the border of transmission zones, which were once free of these diseases with human populations less immune and more receptive. The impact of climate change is also more devastating because of the unpreparedness of Public Health systems to provide adequate response to the events, even when climatic warning is available. Although evidences are strong at the regional and local levels, the studies on impact of climate change on vector-borne diseases and health are producing contradictory results at the global level.

**Conclusions:**

In this paper we discuss the current state of the results and draw on evidences from malaria, dengue and other vector-borne diseases to illustrate the state of current thinking and outline the need for further research to inform our predictions and response.

**Electronic supplementary material:**

The online version of this article (10.1186/s40249-019-0565-1) contains supplementary material, which is available to authorized users.

## Multilingual abstracts

Please see Additional file 1 for translations of the abstract into the five official working languages of the United Nations.

## Background

“Climate is traditionally defined as the description in terms of the mean and variability of relevant atmospheric variables such as temperature, precipitation and wind. Climate can thus be viewed as a synthesis or aggregate of weather.” according to Goosse et al. [1]. These atmospheric parameters are of primary importance for the development and natural life of all ecosystems on our planet, but their influence separately or in combination is highly complex and variable. For this reason, in most situations, we can only approach this understanding through modeling [2]. Correlative models can add an understanding of which parameters are the most important ones in some special regions to explain the climate suitability for a vector or a host. Further, mechanistic models to describe the biological/ecological processes of the transmission are needed and are depending on detailed experimental work to study these processes, and parameters [3]. The climate of the different regions of the world is represented by averages over many years and has been classified into several groups with strong characteristics. Changes in the natural climate can occur at different time-scale, from tens to thousands of years. However, since we can report the climatic variables such as temperatures and precipitations more precisely in a time-based way (daily, weekly, monthly etc.) data show changes including the increase of temperatures, increase and decrease of precipitation and abnormal occurrence of extreme events [4]. How these changes will affect human health and the transmission of vector-borne diseases (VBDs) specifically is a question of great concern [5]. VBDs can be considered as whole ecosystems that include the vectors-pathogens-hosts relationship, linked to specific environmental conditions [6]. The changes in temperature and precipitation, either in intensity, mean, minimum and maximum values, as well as the duration and the variability of the changes, will affect the environment in which the VBDs are transmitted. These environments may become more or less favorable to the vectors and/or the animal reservoirs, as well as disease transmission. The changes will also affect the human host by displacing populations due to drought or flooding, or by affecting agricultural practices and housing systems. The changes will also affect the bionomics of the vectors, in particular the insect vectors.

Over the past 50 years, the earth’s climate has been affected by global warming, with an increase of surface, air and ocean temperature, resulting in the melting of glaciers and the rise of sea levels [5, 7]. The increasing occurrence of extreme events hits most dramatically the poorest countries, already facing the worst infectious diseases situations [8]. The consequences of the climatic changes on public health are not fully understood. However, the risk of emergence of new transmission zones, including in developed countries, is great and emphasizes the need to build our preparations to face such events [9]. Although the causes and consequences of climate changes remains the subject of important discussions [10–12], there is a consensus on the necessity to develop and use new tools for more accurate prediction of the impact of the climatic variables on the different aspects of VBDs transmission [13]. In this paper, evidence will be provided on the impact of climate changes on vectors and VBDs transmission in different types of systems. However, some basic information on how the vectors are responding to temperature change is essential to a better understanding of the impact of the changes.

The insect vectors that transmit pathogens become infectious mainly after the ingestion of the pathogen through a blood meal on an infected host, followed by the pathogens amplification/circulation in the insect’s body, before the vector become infectious [14]. In the case of mosquitoes and arboviruses transmission, the duration of this amplification is called the Extrinsic Incubation Period (EIP) and is strongly dependent upon temperature [15, 16]. Consequently, the climatic conditions and fluctuations have a direct impact on the transmission of arboviruses. The vector competence is a key-factor that may allow or not the transmission and is genetically determined and climatically modulated [17]. Furthermore, insects are cold-blooded or poikilothermic organisms and cannot regulate their own temperature. Since specific body temperatures need to be reached to achieve essential biochemical reactions, the development and physiological functions of the insect is dependent upon the ambient temperature and requires a certain amount of heat to be completed [18]. The amplification of viruses into the mosquito body includes several physiological processes, unknown for many of them [19], but also related to temperature and heat accumulation [20]. The physiological processes in virus amplification start above a threshold temperature and are completed when the thermal constant is reached [21]. The measure of accumulated heat or thermal constant is well described by the physiological time concept and can be expressed through the degree-days method [22–24]. The numerous studies on the Extrinsic Incubation Period (EIP) examining constant and variable temperatures have shown that EIP decreases when temperature increases from a threshold until a maximum, above which the EIP increases again [25, 26]. The impact of temperature changes on VBDs transmission can further have indirect effects such as the biting behavior, fecundity and survival of the vectors [27] and insecticide resistance [28].

Other climatic variables that strongly influence VBDs transmission are water-related, such as rainfall period, duration and abundance, and the humidity of the environment [29]. Although our knowledge of the physiological responses of the insect vectors to the climatic data is increasing, the evidence for changes in transmission are not easily found, both because they require inter-disciplinary studies in regions lacking the necessary expertise, and also because of a lack of adequate data on relationships between climatic variables and transmission parameters such as vectors bionomics including the biting behavior, the resting time for eggs maturation and many other life traits. Nevertheless, the studies presented below clearly show how climate change had an impact or is influencing VBDs transmission for some important diseases, such as malaria, dengue and other diseases in different geographical areas. The objective of this manuscript is clearly to provide available evidences on facts, consequently potential changes based on modelling from future scenarios are not included and discussed.

## Main text: a look at the evidence

### Impact of climatic variables on VBDs transmitted by mosquitoes

To better understand which climatic variables are affecting the VBDs ecosystem and how, the literature was searched firstly on PubMed with the words “climate” and “mosquitoes” and “diseases”, than the word “diseases” was replaced by the name of specific diseases such as “malaria”, “dengue” and other diseases names. From all publications available, only those presenting direct evidences which were facts and situations with proof of relationships between vectors/diseases outcomes and climate changes, were selected to be included in the discussion. From the selected literature, further publications were sometimes referenced. The objective of this review was not to be exhaustive in referring to all existing direct evidences, but more to show which mechanisms were at work and how the changes/trends of the climatic variables are currently influencing the different vector-borne diseases systems. As an example, the cycle of a mosquito-transmitted pathogen is divided into two parts, one part is in the vector and the other part is in the host(s). In the host, the pathogen will find stable and suitable temperature conditions, since the host is regulating its own temperature. On the other hand, in the vector, the pathogen will find the suitable temperature conditions only if the mosquito vector is exposed to a favorable environment. As an example, the *Aedes aegypti* mosquitoes will amplify and transmit dengue viruses only if exposed to temperatures within the range of 20 to 35 °C [30]. These suitable temperature conditions are one of the factors that may explain why the presence of a competent species for transmitting a pathogen is not sufficient for disease transmission, even if the pathogen is introduced in a new region. To better illustrate this, the numerous imported cases of dengue, chikungunya and Zika viruses in European countries from travelers do not result in frequent local transmission, even when the competent vector *Ae. albopictus* is present and active [31]. Further, the vector competence which is the intrinsic ability of a species to amplify and transmit the pathogen is mostly genetically determined and results from long co-evolution between the vector and the pathogen [32]. This competence is often confused with the vectorial capacity, which is the force of infection of a VBD in a host population [33]. Vector competence can be studied in the laboratory under forced climatic conditions and provides the basic understanding of the potential risks of transmission. On the other hand, the vectorial capacity can be estimated only from field conditions with specific parameters and provides an estimation of the real risks of transmission.

When environmental conditions are changing because of climate change, the genetically determined vector competence will not be affected, but the vectorial capacity may dramatically change and provide conditions that are more favorable to outbreak transmission. The vectorial capacity is a function of vector density, which is strongly related to rainfall patterns in the case of mosquitoes [34], of vector survival related to temperature and humidity [35], of the EIP also related to temperature, and of the biting behavior which was found to be both genetically determined and temperature-dependent [36]. None of these parameters fluctuate in the same way, making predictions very unreliable without a complete understanding of the relationships between each parameter and climatic data. However, in some situations, a trend dominates and the impact of climate on a VBD transmission can be determined. Further, since the insects cannot regulate their own body temperature, they are known to look for favorable micro-climatic conditions [37], which mean that the easily available outdoors meteorological data do not represent the true conditions to which the vectors are exposed. As an example, when female mosquitoes are resting in cool and humid places, they are not exposed to high temperatures and dry environments [38]. Consequently, the true understanding of how climatic changes are affecting VBDs is not easily achieved and subject to controversial hypothesis. Nevertheless, the evidence of impact on malaria, dengue and other VBDs presented in this paper should raise awareness and support the need of action to mitigate these effects.

### Evidence of the impact of climate change on malaria vectors and malaria transmission

One of the first pieces of evidence relating climatic warming to an increase in malaria incidence was reported from Rwanda in 1994, showing that an increase in the mean minimum temperature explained 80% of the variance in the monthly malaria estimates in high altitude areas [39]. This finding is consistent with the threshold effect of lower daily temperatures on the extrinsic amplification period. In the following years, malaria transmission was widely used as a model to study the potential effect of different climate scenarios on distribution and patterns of this disease [40–42]. In most of the models, an increase of malaria transmission was predicted under the current rate of global warming, but some models showed a decrease in malaria transmission, due to reduced overall vectorial capacity [43]. Nevertheless, the number of studies reporting the true impact of climate change on malaria is rapidly increasing. Warmer temperatures were found to affect malaria trends in highland regions of East Africa [44], with real changes larger than the predicted ones, probably due to concomitant effects of unknown factors. This evidence shows how difficult it is to assess the real impact of temperatures changes. Warmer temperatures are particularly affecting the Anopheles vectors distribution, such as the distribution of *Anopheles arabiensis* in the slope of Mount Kilimanjaro, resulting in a subsequent change in malaria distribution [45]. The same effect on vector distribution was also found for seven *Anopheles* species in Iran [46] and for *An. gambiae* in Madagascar [47]. To confirm these findings, an increase of malaria incidence at higher altitudes was reported in other countries such as Colombia and Ethiopia [48]. Variations in malaria incidence were also reported during the colder phases of the climatic phenomenon of la Niña in Venezuela [49]. In temperate regions moderately affected by malaria transmission, the effect of changing temperature is more complex. For example, in China malaria transmission is more sensitive to minimum temperatures under cooler climates and to maximum temperatures under warmer climates, with a longer lag effect in cool climate [50]. Consequently, an increase of minimum temperatures will increase malaria incidence in the northern parts, and concomitantly an increase in maximum temperatures will decrease malaria incidence in the southern parts.

Rainfalls and extreme flooding have also been found to have an impact on malaria transmission such as in Uganda, where an extreme flooding event resulted in an increase of malaria risk of 30% [51]. In Zambia, an increase of malaria incidence was correlated to unusual rainfalls between 2008 and 2010 [52] and in Papua New Guinea, the seasonality of malaria was related to the rainfall in two different patterns according to the region. A decrease of rain was associated with a decrease in malaria incidence in the southern coastal region, and at the opposite associated with an increase of malaria incidence in higher altitude [53]. Further, malaria trends in Papua New Guinea were associated to climatic factors at a very local scale with a great variability between locations [54]. Some concomitant effects of temperatures and rainfall were reported from Baringo county in Kenya, where an increase of rainfall was associated with an increase of malaria with a 2-month time lag, and an increase of maximum temperatures was also associated with an increase of malaria with a one-month (or less) time-lag [55].

The moving distribution of malaria vectors, as well as the fluctuations in malaria incidence are challenging vector control activities and impacting upon the malaria elimination targets in some countries. The evidence of changing patterns in malaria affected areas are not easily correlated to climate changes alone, since they take place in an overall changing situation, with modifications of land-use, water-management and human activities exposing different populations to different transmission patterns [56]. Further the potential impact of climate change on current vector control tools has not been properly studied and observations of changes in sleeping behavior when temperatures are rising at night time could have more impact on transmission patterns than the vector-related parameters. However, the evidence reported here clearly shows that climate change is affecting malaria transmission in different ways, challenging already fragile Public Health Systems and putting the human population at greater risks of outbreaks.

### Evidence of impact of climate change on dengue trends

The monitoring of how climate changes are affecting some vector-borne diseases has not been performed systematically over long periods of time [57]. This is particularly true for dengue. However, some evidence has been collected in recent years, showing relationships between temperatures and rainfall changes and dengue transmission patterns. Dengue disease is mostly urban and transmitted mainly by the mosquito species *Ae. aegypti*. Urban temperatures are changing in a drastic way due to the warming climate and consequently they are enhancing, among other factors, dengue transmission and outbreaks, due to higher diurnal temperature range [58]. The combination of urban dynamics and climate change has been well studied in Singapore, where it was estimated that the increase of dengue incidence over the past 40 years, from less than 1000 cases in the 1980s to more than 14 000 cases in 2005, was due to population growth for 86% of the model and to an increase on temperatures for the remaining 14% of the model [59]. This result clearly shows that even without population growth, an increase of temperature may result in an increase in dengue incidence. As previously seen for malaria transmission, the increase of the lower mean temperatures can be linked to an expansion of dengue transmission at higher altitudes and dengue incidence has recently increased in the mountainous country of Nepal [60] The first dengue cases were reported in 2006, followed by an outbreak in 2010, and the last epidemic was reported in late 2017, still ongoing in early 2018. The main city of Kathmandu which is above 1300 m is now affected by dengue outbreaks. In another region, further evidence was collected in Puerto Rico on the impact of increasing temperature on dengue incidence. An increase of 1 °C of the Sea Surface Temperatures (SST) was correlated to an increase of dengue transmission by a factor of 3.4 for the period 1992–2011 [61], and since warming for SST and air surface temperatures (AST) are now evident, a further increase in dengue incidence is expected.

In Vietnam a similar study looking at the impact of rainfall and increased humidity on dengue in the northern coastal city of Haiphong showed that dengue outbreaks are correlated to an increase of both climatic parameters. For each 50 mm rainfall increase and 1% humidity increase, the risk of dengue outbreak increases of 1% [62]. These results are raising great concerns about the current changing patterns of climate in Vietnam, in particular in urban settings [63]. Interestingly, a strong decrease in rainfall followed by drought in Australia is also related to an increase in densities of *Ae. aegypti* mosquitoes, because of increased water storage [64]. This is a secondary effect of climate change linked to human behaviors. In Manila, that has a more tropical environment, dengue was correlated again to rainfall patterns only, with no impact of temperature variations [65]. Other climatic events were also found to be related to dengue cases, with mixed impact of increased temperatures and rainfall, due for example to the El Nino phases in Colombia enhancing dengue transmission [66]. Extreme events such as a tropical cyclone was associated with an increase of dengue incidence in four provinces of China [67]. The impact of climate change on dengue transmission can be more striking on the fringe of different climatic zones, as already mentioned for malaria. The increase of dengue incidence and expansion in Brazil was associated among other factors to climate changes in border areas between endemic and less affected areas [68]. The changes in climate patterns are making these areas more unstable for dengue transmission, with strong impact on Public Health Systems that have to regularly update the dengue transmission maps.

### Evidence of impact of climate change on other vector-borne diseases

Other VBDs diseases are affected by climate change and the example of the human African trypanosomyiasis (HAT) or African sleeping sickness is very informative. This disease is linked to the presence of the tsetse flies vectors, which are very reactive to temperatures and rainfall patterns. The decrease in rainfall in the sahelian border of Western Africa since the 1950s has led to the displacement of the tsetse flies to the southern parts within the 1200 mm rainfall limit per year. Consequently the HAT has also moved from north to south and most of the remaining foci of HAT in the 2000s were found in the southern countries such as Ivory Coast, Ghana and Liberia [69]. Further, in a single country such as Burkina Faso, this shift from north to south could be measured and was estimated to be between 25 and 150 km, with an estimated reduction of the tsetse belt of about 70 000 km^2^ [70]. This strong decrease of the favorable environment for tsetse flies and HAT was attributed to both climate change, with severe droughts impacting not only the vectors but also the human distribution, and a strong human population growth modifying the tsetse habitats. A secondary and interesting effect of the climate change on the tsetse flies vectors is the fragmentation of the tsetse habitat which has an impact on flies dynamics and further reduce their densities [71]. More recently in another part of Africa, the increase of temperature was associated to the drastic decline in the tsetse flies densities in the Zambezi Valley [72], with a displacement of the vector populations to higher altitude areas (such as already seen in anopheles vectors and malaria) that are thus becoming more favorable to disease transmission. For another parasitic disease, the Leishmaniasis, transmitted by the sand flies, a shift in vectors distribution has been reported from south to north in Europe which is attributed to changes in the climatic conditions as modeled through an ecological niche approach [73]. Again, sand flies species competent for transmitting Leishmania parasites were recently found for the first time in Belgium and Germany, creating new risks of transmission in countries that are currently free of disease transmission. The same displacement of competent sand flies was also reported in the southern hemisphere, from north to south Argentina due to an increase of temperatures in more temperate regions of this country. This displacement was associated with new local cases of cutaneous leishmaniasis cases in the outskirts of the very populated city of Cordoba [74]. Consequently, the risk of extending the current transmission area of this disease in Argentina is very serious, and can be link to the climate change. In the case of leishmaniasis, the change in sand flies distribution due to climate change can also have consequences on the elimination program such as in Nepal for elimination of visceral leishmaniasis (VL). Between 1999 and 2009, 11 additional districts situated in mountains areas are reporting VL cases [60], and the country has now the obligation to extend the elimination program to newly affected areas, with all the costs and logistics issues for a low income country. The impact on climate change on VBDs transmission can be seen not only on the vectors, but also on the host such as in the Plague ecosystems, as demonstrated in the Daurian ground squirrel and Mongolian gerbil [75]. The first host densities are positively associated to vegetation, link to high temperatures and rainfalls. In the contrary the second host densities are negatively associated with vegetation. As a consequence, in the current climate change situation, the surveillance of plague foci in their natural environment is driven by the host behavior, which is very different for the 2 hosts, and a proper monitoring of the plague circulation with associated risks of emergence as human disease will now take into account the host densities link to vegetation link to climate change. Extreme events which are one the most important outcomes of the climate change trends have consequences on VBDs transmission other than malaria and dengue, such as in the case of Rift Valley Fever Virus (RVFV). The impact of the droughts and El-Nino/Southern Oscillation (ENSO) events on the RVFV transmission has been well described, with rainfalls anomalies leading to the emergence of huge densities of vectors and outbreaks in the livestock and extension of the disease to humans, resulting in a double impact on very vulnerable human populations that are losing their livestock and facing the disease [76]. The improved current knowledge on the dynamics of this RVFV transmission patterns associated to climate events as well as community behavior is now allowing the prevention and mitigation measures [77].

## Conclusions

Although it is not fully understood how climatic variables, changes in trends, extreme events and climate variability are directly affecting the transmission of vector-borne diseases, much evidence can be found to confirm that increasing temperatures due to global warming have an impact on these diseases. This evidence includes contrasting effects with increasing disease incidence in some situations and decreasing disease incidence in other situations. The same effects are also reported for livestock diseases, which have been studied more intensively because of their economic outcomes. For example, the increase in temperature is related to the expansion of some vectors and consequently of the diseases they transmit such as *Culicoides imicola*, transmitting the bluetongue virus, and on the contrary are also related to low survival of tsetse flies and a subsequent decrease of animal trypanosomiasis [76]. The consequences of rise of temperatures are thus not a one direction impact, and the VBDs ecosystems are responding in different and sometimes opposite ways. These findings reinforce the necessity to look at these changes with local and disease specific approaches.

One of the most common effect of climate change on VBDs is the change in vector and disease distribution found not only for malaria and dengue, but also for other diseases such as HAT, Leishmaniasis and the Japanese Encephalitis, now emerging as a human and livestock disease on the slopes of the Himalayan highlands [78]. Even in temperate regions, the displacement of VBDs has been reported and in Canada heatwaves were found to be related to the northern displacement of Lyme disease [79]. For Lyme disease, global warming has resulted in the tick vectors finding suitable conditions in northern areas, and also on the animal reservoir of *Borrelia burgdorferi* which are migrating northward and creating favorable conditions for Lyme disease transmission to extend to northern locations [80]. These changes in geographical distribution and expansion of the diseases will result in strong effects on the human and/or animal populations that are naïve to the disease.

The impact of climate changes on VBDs is complex and the occurrence of opposite effects makes general predictions almost impossible. In order to provide recommendations, based on evidences that can be transformed into policies the impact of climate change has to be investigated at the very specific and local scale. Further integrated approaches are needed because of several confounding factors, which include the host behavior and human population dynamics (growth, mobility, …). Nevertheless, the consequences of these changes already have an impact on Public Health, and the health systems need to be prepared to face epidemics and to mitigate these threats. Preparedness should be based on multi-sectorial concepts and framework, include a deeper understanding of the biological phenomenon as well as a plan for strengthening health systems to respond to different levels of emergency. The development of mitigation measures is needed at all levels, from the global to the local and should co-ordinate and take advantage of push to achieve the Sustainable Development Goals [81].

## Additional file

Additional file 1: Multilingual abstracts in the five official working languages of the United Nations. (PDF 378 kb)

## Data Availability

The authors declare that all material and data used in the manuscript are referenced and freely available online.

## Abbreviations

AST: Air surface temperatures
EIP: Extrinsic incubation period
ENSO: El-Nino/Southern Oscillation
HAT: Human African trypanosomiasis
RVFV: Rift Valley fever virus
SST: Sea surface temperatures
VBDs: Vector-borne diseases
VL: Visceral leishmaniasis
