# Supplementary material for: Impact of past and on-going changes on climate and weather on vector-borne diseases transmission: a look at the evidence
Source: Infect Dis Poverty. 2019 Jun 13;8:51. doi: 10.1186/s40249-019-0565-1 (PMC6567422; doi:10.1186/s40249-019-0565-1)
Supplement: Supplementary file 1 — Multilingual abstracts in the five official working languages of the United Nations. (PDF 378 kb) [file 40249_2019_565_MOESM1_ESM.pdf]

Translation of the abstract into the five official working languages of the United Nations

الادله علي نظره :بالنواقل المنقولة الامراض انتقال علي والطقس المناخ في والمستمرة السابقة التغيرات اثر

ريدر سي جون و فاي فلورنس

مجرده
درجه أساسا هي المنقولة للامراض الايكولوجيه النظم علي مباشرا تاثيرا تؤثر التي المناخية المتغيرات :الخلفية معظم لان أيضا ولكن ,المتغيرات هذه علي بشده تعتمد بيونوميميكس ناقلات لان فقط ليس هذا .والامطار الحرارة التغيرات اثر فهم السهل من وليس .الممرض والتضخيم والتنمية المضيف السلوك مثل ,تتاثر الانظمه عناصر ما غالبا ، ذلك علي وبناء .معا تعمل المربكة العوامل من العديد لان ، الامراض هذه انتقال أنماط علي المناخية الادله بعض علي العثور يمكن ، ذلك ومع .رياضيه نماذج من مستمده فرضيه إلى الآثار بهذه المعرفة تستند .بالنواقل المنقولة الامراض من للعديد المباشرة
من والعديد ، الضنك حمي مثل الفيروسات وامراض ، للملاريا متاحه المناخ تغير تاثير علي الادله :الرئيسية الهيئة البشرية المثقبيات والداء ، الياباني الدماغ والتهاب ، المتصدع الوادي مثل الأخرى والفيروسية الطفيلية الامراض لتفشي الرئيسي السبب هو المتطرفة الاحداث وكذلك والامطار الحرارة تغير اثر ان تبين وقد .والشمانيا الافريقيه التوزيع علي بقوة المناخ يؤثر ، الرئيسية القيادة عوامل بين ومن .العالمي المجتمع لدي الانزعاج ويثير الامراض في ، المناخ تغير إلى ينظر ، ذلك علي وعلاوة .المناخ تغير بسبب بسرعة تتغير التي ، الحشرات لنواقل الجغرافي أكبر بشكل للنظر لافت بشكل بالنواقل المنقولة الامراض علي يؤثر انه علي ، السواء علي المباشرة والادله النماذج .الامراض هذه من خاليه كانت والتي ، الإرسال مناطق حدود في تكون ما غالبا التي المختلفة المناخية المناطق في استجابه لتوفير العامة الصحة نظم استعداد عدم بسبب تدميرا أكثر المناخ تغير اثر ان كما .تقبلا وأكثر مناعة اقل الإقليمي الصعيدين علي قويه الادله ان من الرغم وعلي .متاحا المناخي الإنذار يكون عندما حتى ، للاحداث كافيه نتائج عن تسفر والصحة بالنواقل المنقولة الامراض علي المناخ تغير بتاثير المتعلقة الدراسات فان ، والمحلي .العالمي الصعيد علي متناقضة
الضنك وحمي الملاريا من المستمدة الادله علي والاعتماد للنتائج الراهنة الحالة الورقة هذه في نناقش :الخلاصة لتنوير البحوث من مزيد إلى الحاجة وتحديد الحالية التفكير حاله لتوضيح بالنواقل المنقولة الامراض من وغيرها .استجابه توقعاتنا

Translated from English version into Arabic by Hamza Shaffison,

既往及持续的天气和气候变化对于媒传疾病传播的影响：基于证据的综述

Florence Fouque and John C Reeder

摘要：

引言：温度和降雨量是直接影响媒传疾病生态系统的主要气候变量。这些变量不仅直接影响病媒的生态学特征，而且也对生态系统中宿主行为和发育以及病原体的扩增等诸多因素也会造成影响。由于多种因素交织，研究气候变化对疾病传播模式的影响相对困难。通常基于数学模型对假说进行推演来了解这些因素的作用。然而，多种媒传疾病可以为我们提供一些直接的证据。

正文：从疟疾、登革热等虫媒病毒和裂谷热、日本脑炎、人类非洲锥虫病和利什曼病等许多寄生性或病毒性疾病中可以找到一些气候变化影响的证据。温度、降雨量的改变以及极端事件的发生是疾病暴发的主要原因，并为全人类敲响了警钟。在主要驱动因素中，气候变化强烈影响病媒的地理分布并使其随之快速变化。一些模型和直接证据表明，气候变化在媒介传播区域边缘影响更为显著，这些区域未曾出现过相关疾病，人群缺乏免疫力并易感。即使发布气候预警，由于当地卫生系统准备不足无法正确应对，气候变化造成的影响更为严重。虽然区域性和地区性提供的证据较多，但在气候变化对全球媒传疾病和人类健康的影响研究尚不充足。

结论：我们讨论了研究现状并从疟疾、登革热以及其它媒传疾病中找到了一些证据来说明目前的研究状态，并提出有必要进行更深入的研究来为我们的预测和应对行为提供信息。

Translated from English version into Chinese by Peng Song, edited by Jin Chen

Impact des changements climatiques et météorologiques passés et actuels sur la transmission des maladies vectorielles : examen des données probantes

Florence Fouque et John C Reeder

Résumé

Contexte : Les principales variables climatiques qui influencent directement les écosystèmes des maladies à transmission vectorielle sont la température et la pluviométrie, non seulement parce que la bionomie des vecteurs dépend fortement de ces deux variables, mais aussi parce qu’elles influent sur la plupart des éléments des systèmes, comme le comportement et le développement des hôtes ainsi que l’amplification des pathogènes. L’impact des changements climatiques sur les schémas de transmission de ces maladies est difficile à appréhender car de nombreux facteurs de confusion agissent conjointement. Par conséquent, la connaissance de ces effets repose souvent sur des hypothèses basées sur des modèles mathématiques. Certains effets directs peuvent néanmoins être constatés pour plusieurs maladies à transmission vectorielle.

Section principale : Il existe des données prouvant l’impact du changement climatique sur le paludisme, les arboviroses telles que la dengue, et de nombreuses autres maladies parasitaires et virales comme la fièvre de la vallée du Rift, l’encéphalite japonaise, la trypanosomiase humaine africaine et la leishmaniose. L’incidence des changements de température et de pluviométrie, d’une part, et d’autre part, les événements extrêmes, sont apparus comme les causes principales des épidémies et inquiètent la communauté internationale. Parmi les principaux facteurs d’influence, le climat a un effet important sur la répartition géographique des insectes vecteurs, qui change rapidement en fonction des variations climatiques. Par ailleurs, aussi bien pour les modèles que pour

les effets directs, on constate que le changement climatique a une incidence plus forte sur les maladies vectorielles en périphérie de différentes zones climatiques, souvent à la limite des zones de transmission, dans des régions qui étaient jusque-là exemptes de ces maladies et dont les populations humaines étaient moins immunisées et plus réceptives. L'impact du changement climatique est encore plus dévastateur lorsque les systèmes de santé publique ne sont pas prêts à répondre de façon adéquate aux événements, même en cas d'alertes préalables sur des catastrophes climatiques. Malgré les solides données probantes relevées au niveau régional et local, les études sur l'impact des changements climatiques sur les maladies vectorielles et la santé donnent des résultats contradictoires au niveau planétaire.
Conclusion : Nous dressons dans le présent article un bilan des résultats en nous appuyant sur des données concernant le paludisme, la dengue et d'autres maladies à transmission vectorielle, afin d'illustrer l'état actuel des réflexions et de mettre en lumière la nécessité de mener de nouvelles recherches pour étayer nos prédictions et définir la réponse à apporter.

Translated from English version into French by Suzanne Assenat, Revised by Estelle Rio, through

TRANSLATORS WITHOUT BORDERS

Влияние климатических и природных изменений в прошлом и в настоящем на передачу трансмиссивных болезней: анализ доказательств

Флоренс Фуке и Джон Си Ридер

Аннотация

Предпосылки:климатические изменения, которые <напрямую влияют на экосистемы трансмиссивных болезней-это главным образом температура и осадки. Причина не только в том, что экологические факторы передачи заболеваний напрямую связаны с этими изменениями, но и в том, что большинство элементов данных систем подвергаются воздействию, как организм-носитель, развитие и патогенная амплификация. Связь между климатическими изменениями и случаями заражения данным заболеваниями понять нелегко ввиду взаимодействия множества мешающих факторов. Следовательно, сведения о воздействиях климатических изменений часто основаны на гипотезах, которые выведены из математических теорий. Однако, можно найти некоторые прямые доказательства для некоторых трансмиссионных болезней.
Основная часть: доказательства влияния климатических изменений прослеживаются на примере малярии, арбовирусных инфекций, таких как лихорадка денге, и многих других паразитических и вирусных заболеваний, как лихорадка Рифт-Валли, японский энцефалит, африканский трипаносомоз и лейшманиоз. Влияние температурных изменений и осадков, а также экстремальные условия, оказались главными возбудителями вспышек заболеваний и вызывают угрозу для мирового сообщества. Из главных движущих факторов климат оказывает существенное влияние на географическое распространение насекомых-

возбудителей, которое быстро изменяется из-за климатических изменений. Кроме того, согласно обеим моделям и прямым доказательствам, изменение климата является главным фактором распространения трансмиссивных болезней в различных климатических зонах, зачастую на границе ареалов передачи, которые когда-то не были поражены данными инфекциями и где люди менее иммуноустойчивы и более восприимчивы к ним. Воздействие климатического изменения еще более разрушительно в виду неготовности систем здравоохранения дать адекватный отпор происходящим событиям даже при наличии климатических угроз. Несмотря на то, что на региональном и местном уровнях доказательства являются убедительными, исследования влияния изменения климата на трансмиссивные болезни и здоровье человека дают противоречивые результаты на глобальном уровне.
Вывод : в данной статье мы рассматриваем текущие результаты и опираемся на доказательства влияния климатических изменений на малярию, лихорадку денге и другие трансмиссивные болезни, для того, чтобы продемонстрировать текущую позицию и подчеркнуть необходимость проведения дальнейших исследований, дабы сообщить о наших прогнозах и реакции.

Translated from English version into Russian by Gunel Huseynbayova, Revised by Alexander Somin, through

TRANSLATORS
WITHOUT BORDERS

El impacto de los cambios climáticos y meteorológicos pasados y en curso sobre la transmisión de enfermedades transmitidas por vectores

Florence Fouque y John C. Reeder

Resumen
Antecedentes: las variables climáticas que influyen de manera directa en los ecosistemas de las enfermedades transmitidas por vectores son principalmente la temperatura y las precipitaciones. Esto se debe no solo a que la bionomía de los vectores depende en gran medida de estas variables, sino también a que la mayoría de los elementos de los sistemas se ven afectados, tales como el comportamiento y el desarrollo de los huéspedes y la amplificación de los patógenos. El impacto de los cambios climáticos en los patrones de transmisión de estas enfermedades no es fácil de comprender, ya que muchos factores de confusión actúan conjuntamente. Por consiguiente, el conocimiento de estas repercusiones a menudo se basa en hipótesis derivadas de modelos matemáticos. Sin embargo, para varias enfermedades transmitidas por vectores pueden encontrarse algunas pruebas directas.
Texto principal: existen pruebas del impacto del cambio climático disponibles para la malaria, las enfermedades por arbovirus, como el dengue; y muchas otras enfermedades parasitarias y virales, como la fiebre del Valle del Rift, la encefalitis japonesa, la tripanosomiasis humana africana y la

leishmaniasis. Se descubrió que el efecto del cambio de temperatura y de las precipitaciones, junto con los fenómenos extremos, son la principal causa de brotes y son el motivo de alarma para la comunidad mundial. Entre los principales factores determinantes, el clima influye en gran medida en la distribución geográfica de los insectos vectores, la cual está cambiando rápidamente debido al cambio climático. Además, tanto en los modelos como en las pruebas directas, se considera que el cambio climático afecta de manera más sorprendente a las enfermedades transmitidas por vectores en el margen de distintas zonas climáticas, a menudo en la frontera de las zonas de transmisión que antes estaban libres de estas enfermedades con poblaciones humanas menos inmunes y más receptivas. Asimismo, el impacto del cambio climático es más devastador debido a la falta de preparación de los sistemas de salud pública para proporcionar una respuesta adecuada ante los acontecimientos, incluso cuando la alerta climática está disponible. A pesar de que las pruebas a nivel regional y local son sólidas, los estudios sobre el impacto del cambio climático en las enfermedades transmitidas por vectores y en la salud generan resultados contradictorios a nivel mundial.

Conclusión: en el presente artículo analizamos el estado actual de los resultados y nos basamos en las pruebas de la malaria, el dengue y otras enfermedades transmitidas por vectores para ilustrar el estado de reflexión actual y para exponer la necesidad de una mayor investigación para informar nuestras predicciones y nuestra respuesta.

Translated from English version into Spanish by Mayra León, Revised by Guillermo Roscoe, through

TRANSLATORS
WITHOUT BORDERS
